# Study of the skin anatomy with high-frequency (22 MHz) ultrasonography and histological correlation*

**DOI:** 10.1590/0100-3984.2014.0028

**Published:** 2015

**Authors:** Elisa de Oliveira Barcaui, Antonio Carlos Pires Carvalho, Juan Piñeiro-Maceira, Carlos Baptista Barcaui, Heleno Moraes

**Affiliations:** 1 Master Fellow degree, Program of Post-graduation in Medicine (Radiology), Universidade Federal do Rio de Janeiro (UFRJ), Rio de Janeiro, RJ, Brazil.; 2 Associate Professor, Department of Radiology, Coordinator for the Program of Post-graduation in Medicine (Radiology), Universidade Federal do Rio de Janeiro (UFRJ), Rio de Janeiro, RJ, Brazil.; 3 Collaborating Professor of Dermatology and Pathological Anatomy, School of Medical Sciences, Universidade do Estado do Rio de Janeiro (UERJ), Rio de Janeiro, RJ, Brazil.; 4 Associate Professor of Dermatology, School of Medical Sciences, Universidade do Estado do Rio de Janeiro (UERJ), Rio de Janeiro, RJ, Brazil.; 5 Associate Professor of Pathological Anatomy, School of Medical Sciences, Universidade do Estado do Rio de Janeiro (UERJ), Rio de Janeiro, RJ, Brazil.

**Keywords:** Ultrasonography, Dermatology, Skin, Histology

## Abstract

The present essay is aimed at getting the radiologist familiar with the basic histological skin structure, allowing for a better correlation with sonographic findings. A high-frequency (22 MHz) ultrasonography apparatus was utilized in the present study. The histological analysis was performed after the skin specimens fixation with formalin, inclusion in paraffin blocks and subsequent staining with hematoxylin-eosin. The authors present a literature review showing the relationship between sonographic and histological findings in normal cutaneous tissue, and discuss the technique for a better performance of the sonographic scan. High-frequency ultrasonography is an excellent tool for the diagnosis of different skin conditions. However, as this method is operator-dependent, it is crucial to understand the normal skin structure as well as the correlation between histological and sonographic findings.

## Abstract (pt)

O objetivo deste trabalho é familiarizar o radiologista com a estrutura histológica cutânea, o que torna possível uma melhor correlação dos achados ultrassonográficos da pele. Para o exame radiológico foi utilizado aparelho de ultrassom de alta frequência (22 MHz). O exame histológico foi realizado após fixação do material em formol, inclusão em parafina e coloração com hematoxilina-eosina. Os autores fazem uma revisão da literatura, demonstram a relação dos achados ultrassonográficos e histológicos do tecido cutâneo normal e discutem a técnica para o melhor aproveitamento do exame ultrassonográfico da pele. O ultrassom de alta frequência representa uma excelente ferramenta no diagnóstico das diferentes alterações cutâneas. Como o método é operador-dependente, é crucial o perfeito entendimento da pele normal e sua equivalência histológica/ultrassonográfica.

## INTRODUCTION

A series of recent studies published in Brazil have highlighted the relevance of ultrasonography in the diagnosis and treatment of several diseases^(1-15)^. Ultrasonography is utilized in dermatology since the 1970's to evaluate skin thickening^(16)^. The development of apparatuses with frequency > 15 MHz has allowed for the identification of the different layers and structures of the skin and adnexal, considerably widening the use of the method in cases of dermatological diseases. High-frequency apparatuses have low penetration and, consequently, excellent resolution for visualization of superficial structures^(17)^.

The skin presents its own characteristics according to the anatomical region, age and race. The knowledge of anatomy and morphology is fundamental for a perfect assessment of the structures observed at ultrasonography^(18)^.

The present essay is aimed at demonstrating the correlation between the sonographic study and histological analysis of the normal skin, facilitating the understanding and the diagnosis of dermatological diseases.

## THE SKIN

The skin is composed of two layers, namely, dermis and epidermis. Because of the proximity and reactional behavior of the subcutaneous tissue in the different pathological processes, some authors consider it as a third layer^(19)^.

Approximately 95% of the epidermis is composed of cells called keratinocytes that synthesize a protein called keratin. Keratinocytes form four layers that undergo continuous transformation. From the bottom to the surface, such layers are the following: basal, spinous, granular and corneous layers. Melanocytes, Langerhans and Merkel cells form the remaining 5%^(18,20)^.

Fibroblasts, dermal dendritic cells, mastocytes and macrophages constitute the main cell components of the dermis. Extracellular components include collagen and elastic fibers, and amorphous fundamental substance. The dermis is divided into two compartments: papillary dermis and reticular dermis. The connective tissue is the most abundant component of this region (70%) and is constituted of collagen fibers. In the papillary dermis these fibers are more delicate and fine as compared with those of the reticular dermis where such fibers present like thicker bundles of fibers^(19)^.

Between the epidermis and the dermis there is the basal membrane zone, a macromolecules mesh that connects the basal layer keratinocytes with the collagen fibers of the papillary dermis^(20)^.

**Figure 1:**
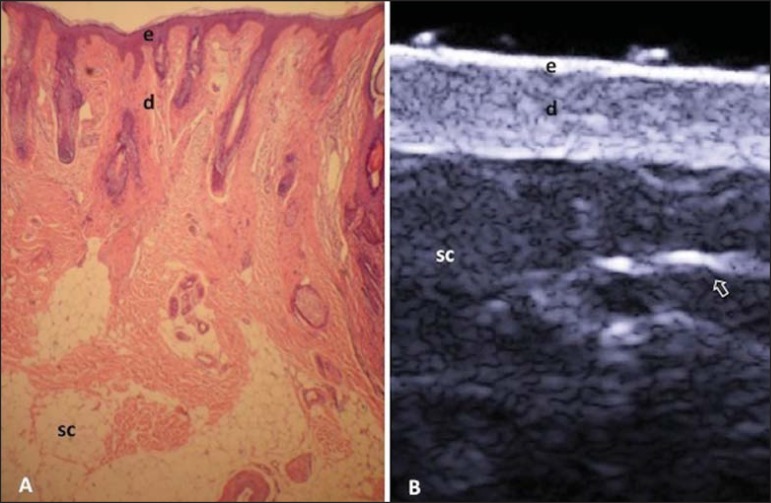
Non glabrous skin anatomy. **A:** Normal skin histology. **B:** High-frequency ultrasonography (HFUS), crosssectional view. Epidermis (e), dermis (d) and subcutaneous tissue (sc) with presence of fibrotic septa (arrow).

The subcutaneous tissue is composed of adipocytes presenting globose cytoplasm without vacuoles. The fat lobules are separated by fibrotic septa crossed by small vessels^(21)^ (Figure 1A).

The echogenicity of each layer depends on its main component: keratin (epidermis), collagen (dermis) and fat lobules (subcutaneous). At the sonographic image, the epidermis is seen as a hyperechoic line, the dermis as a less bright hyperechoic band and the subcutaneous layer as a hypoechoic layer with the presence of hyperechoic fibrotic septa inside^(16)^ (Figure 1B).

**Figure 2:**
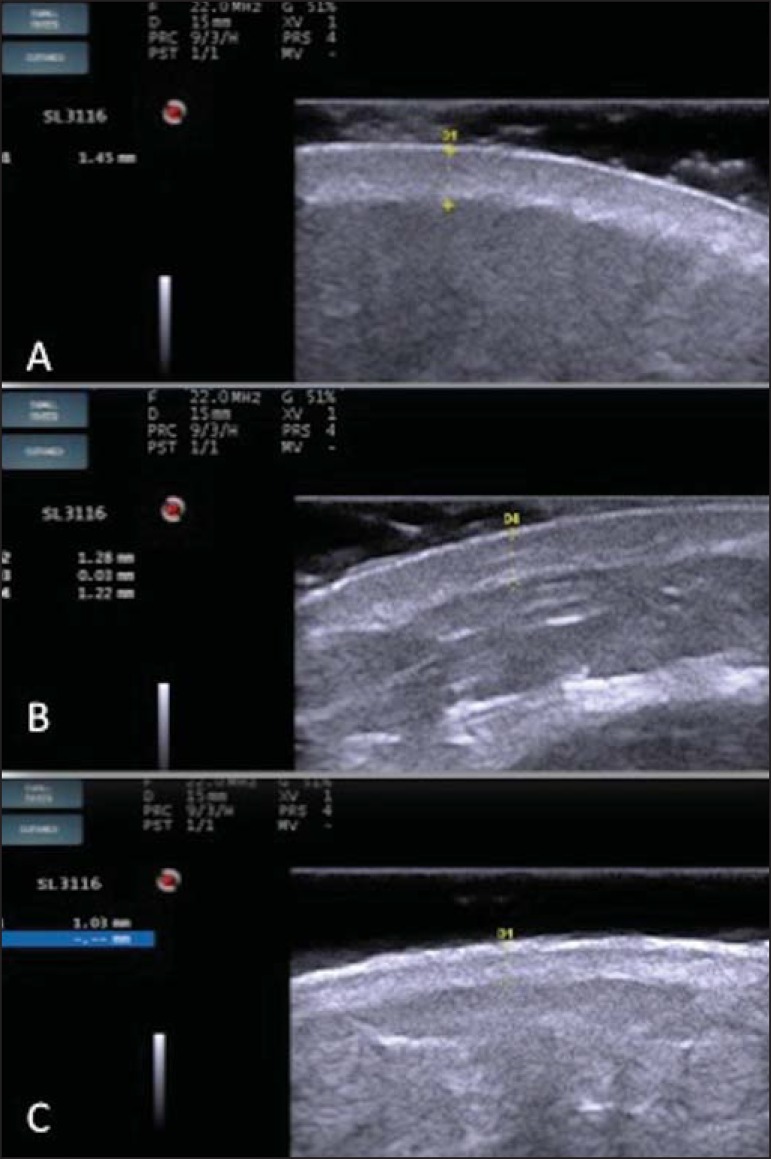
HFUS, cross sectional view, anterior region of the right forearm in consanguineous patients with a single skin phototype. **A:** 3-year-old patient. Thin epidermis and 1.45 mm-thick dermis. **B:** 25-year-old patient. Dermis measuring 1.22 mm in thickness. **C:** 55-year-old patient. Epidermal thickening and 1.03 mm-thick dermis.

**Figure 3:**
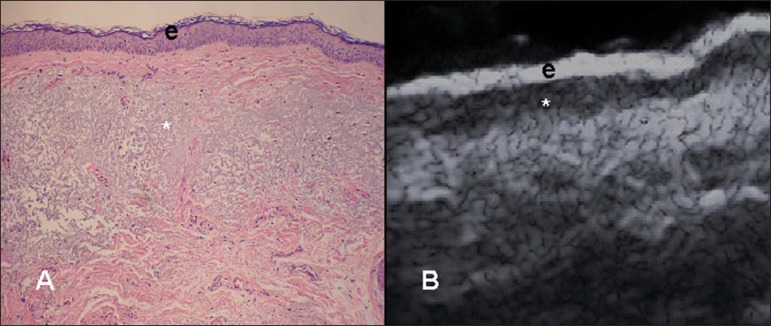
**A:** Histology. Epidermis (e), low-echogenicity subepidermal band corresponding to elastosis present in the connective tissue (asterisk). **B:** HFUS, crosssectional view.

The echogenicity and the thickness of the dermis are variable, according to the patient's age (Figure 2). In neonates, it is slightly hypoechoic. In individuals of advanced age or intense actinic damage, a hypoechoic area called sub-epidermal low echogenic band is observed between the dermis and the epidermis, representing a probable sonographic manifestation of elastosis and edema in the papillary dermis (Figure 3). Some authors propose that the measurement of such a band could quantify the actinic damage^(22)^.

## TOPOGRAPHIC ASSESSMENT

### Glabrous skin - palmo-plantar region

**Figure 4:**
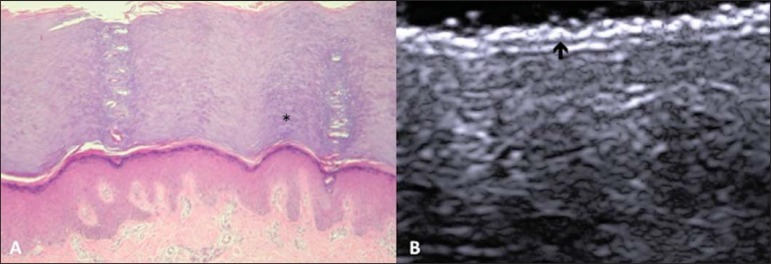
Glabrous skin. **A:** Histology. Thicker corneous layer and presence of stratum lucidum (asterisk). **B:** HFUS, cross-sectional view. Hyperechoic epidermis with bilaminar appearance (arrow).

Histologically, an additional epidermal layer is observed between the granular and corneous layers, called stratum lucidum. The cells of this layer are nucleated and called transitional cells^(18)^ (Figure 4A). At high-frequency ultrasonography (HFUS), the epidermis of this anatomical area is seen as a bilaminar hyperechoic structure^(23)^, possibly resulting from the contrast between the epidermis itself and a very thick and compact stratum corneum. The other skin layers are similar to the non glabrous skin (Figure 4B).

### Scalp and hair shaft

**Figure 5:**
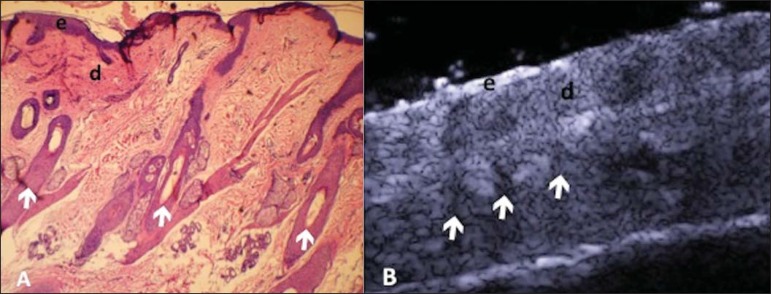
Scalp. **A:** Histology, longitudinal section. **B:** HFUS, longitudinal view. Epidermis (e), dermis (d), oblique hypoechoic bands corresponding to hair follicles (arrows).

The hair follicle is a dynamic microstructure attached to the skin, responsible for the production of hair, in constant self-regeneration. For this reason, it presents a cyclic behavior. Periods of high mitotic activity and cell differentiation (anagenous phase) are interrupted by a remodelling phase (catagenous phase), followed by a quiescent period (telogen phase), with a subsequent growth restart (Figure 5A).

At HFUS, the appearance of the scalp skin layers is similar to the skin of other anatomical sites: hyperechoic line (epidermis), hyperechoic band (dermis) and hypoechoic band (subcutaneous). More deeply, a hypoechoic band corresponding to the galea is observed and, immediately after, the calvarium demonstrated by an intensely hyperechoic line. Longitudinal hypoechoic structures corresponding to the hair follicles are observed on the scalp images. Depending on the phase of the hair growth cycle, such structures are observed at different skin layers, as follows: in the telogen phase, the bulb is located in the dermis, while in the anagenous phase, the bulb is located in the subcutaneous tissue^(24)^ (Figure 5B).

**Figure 6:**
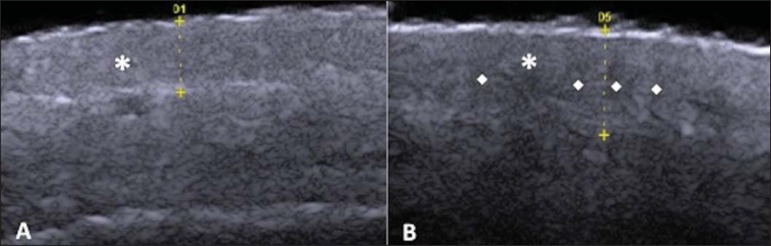
HFUS, longitudinal view, scalp. Male, 41-year-old patient. Variation of the skin thickness and in the number of hair follicles. **A:** Frontal region. **B:** Occipital region. Dermis (asterisks), hair follicle (diamonds).

The scalp skin of the frontal region is thinner than the occipital region, and the hair follicles density is variable^(24)^ (Figure 6).

**Figure 7:**
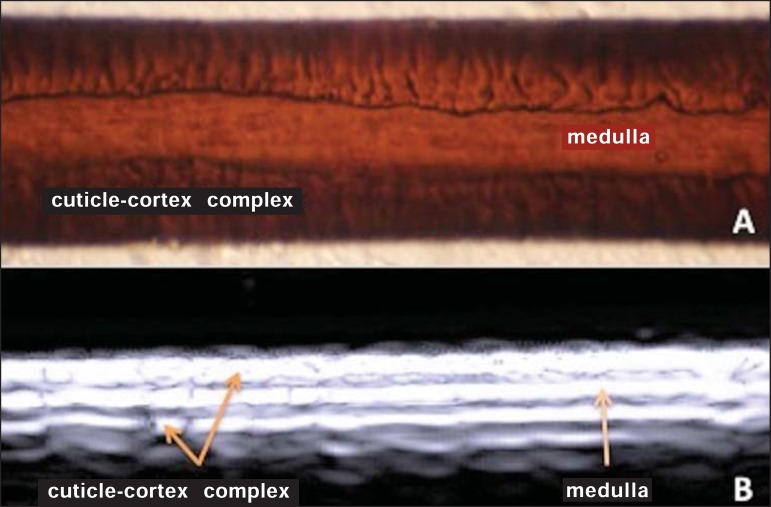
Hair shaft. **A:** Histology. Cortexcuticle- medulla structure. **B:** Longitudinal view, trilaminar.

The anagenous phase of the hair follicle originates the terminal hair shaft composed of cuticle, cortex and medulla^(20)^ (Figure 7A). HFUS, longitudinal view allows for differentiating a trilaminar hyperechoic structure, probably corresponding to the arrangement of keratin layers (Figure 7B).

### Ungual unit

**Figure 8:**
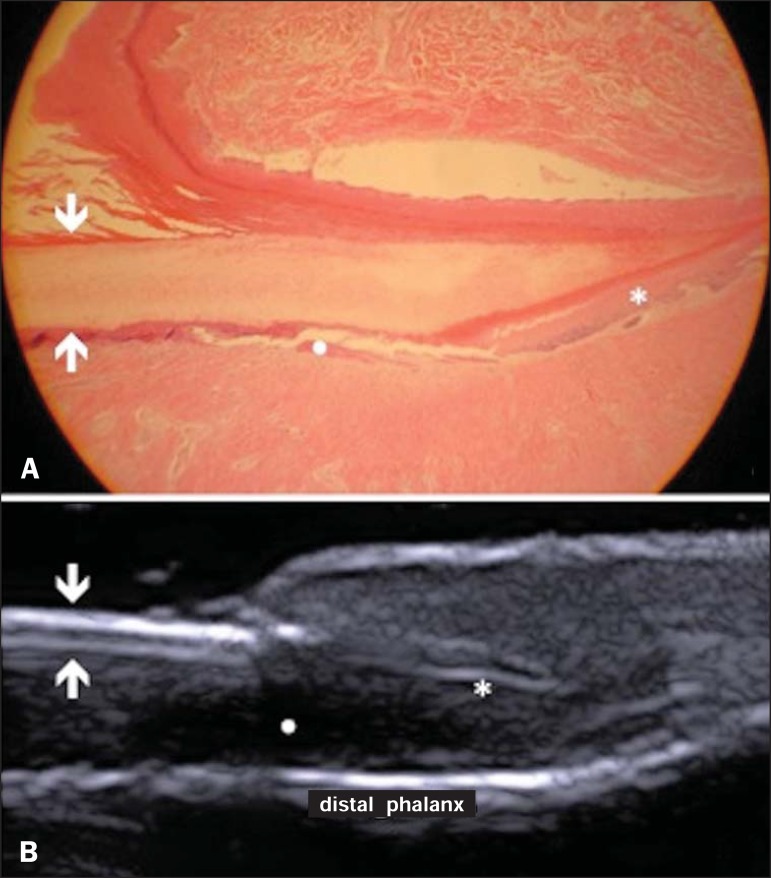
Normal ungual unit, longitudinal section. **A:** Histological section. **B:** HFUS. Ventral plate (downward arrow), dorsal plate (upward arrow), ungual matrix (asterisk), ungual bed (bold circle).

The ungual unit has five components, as follows: matrix, ungual lamina, cuticle, ungual bed and ungual folds (proximal, lateral and distal) ^(19,20)^ (Figure 8A).

At HFUS, the ungual lamina is subdivided into dorsal and ventral plates, generating a bilaminar, hyperechoic aspect separated by a thin hypoechoic line. A hypoechoic ungual bed is beneath the ungual lamina. The echogenic matrix may be observed at the proximal aspect of the ungual bed. A hypoechoic line corresponding to the bone of the distal phalanx is beneath the ungual bed^(18,25)^ (Figure 8B).

### DISCUSSION

The first step for the interpretation of the sonographic findings is the recognition of the different skin structures. In a single individual, it is possible to observe distinctive sonographic patterns, depending on the studied anatomical site.

Apparatuses with frequencies > 15 MHz allow for the study of the skin and adnexa, since the skin layers and structures can be distinguished. However, apparatuses with frequencies > 20 MHz present better imaging resolution for the study of superficial structures.

In the sonographic evaluation of the skin, one recommends the utilization of a thick gel layer between the transducer and the skin surface, in order to obtain a better focal point. A gelatinous cushion may be employed for the study of the ungual unit.

It is important to utilize a delicate transducer, adaptable to the different contour of the body segments such as face and distal phalanx. The contact of the transducer with the skin must be the gentlest possible to avoid compression of the anatomical structures which, in this tissue, are thin and superficial.

The hair on the region to be studied should be preferentially shaved and not cut with a scissor, in order to allow for a better contact between the transducer and the skin. Considering that different scalp diseases develop with alteration of the hair shaft, this can also be evaluated at HFUS.

For the study of dermal lesions with crusts or marked keratinization it is recommended that such abnormalities are removed since they cause acoustic beam attenuation, reducing the accuracy of the examination.

An appropriate skin evaluation utilizing HFUS includes defining the exact region to be studied, differentiate the skin layers, its thickness and vascularization, and identify possible associated pathological findings.

## CONCLUSION

The arrival of HFUS has facilitated a more detailed study of the skin and adnexa, allowing for the diagnosis and definition of the treatment of dermatological diseases, which requires a deeper knowledge of the sonographic aspect of the normal skin.
